# Sustainable Solutions for Sea Monitoring With Robotic Sailboats: N-Boat and F-Boat Twins

**DOI:** 10.3389/frobt.2022.788212

**Published:** 2022-04-05

**Authors:** Alvaro P. F. Negreiros, Wanderson S. Correa, André P. D. de Araujo, Davi H. Santos, João M. Vilas-Boas, Daniel H. N. Dias, Esteban W. G. Clua, Luiz M. G. Gonçalves

**Affiliations:** ^1^ Electrical and Computer Engineering Graduate Program, Universidade Federal Do Rio Grande do Norte, Natal, Brazil; ^2^ Computing Institute, Universidade Federal Fluminense, Niteroi, Brazil; ^3^ Academic Directorate of Information Technology, Instituto Federal de Educação Tecnológica Do Rio Grande do Norte, Natal, Brazil; ^4^ Electrical Engineering Department, Universidade Federal Fluminense, Niteroi, Brazil

**Keywords:** autonomous sailboat, energy self-generation, energy management, renewable energy, boltzman machine

## Abstract

Strategic management and production of internal energy in autonomous robots is becoming a research topic with growing importance, especially for platforms that target long-endurance missions, with long-range and duration. It is fundamental for autonomous vehicles to have energy self-generation capability to improve energy autonomy, especially in situations where refueling is not viable, such as an autonomous sailboat in ocean traversing. Hence, the development of energy estimation and management solutions is an important research topic to better optimize the use of available energy supply and generation potential. In this work, we revisit the challenges behind the project design and construction for two fully autonomous sailboats and propose a methodology based on the Restricted Boltzmann Machine (RBM) in order to find the best way to manage the supplementary energy generated by solar panels. To verify the approach, we introduce a case study with our two developed sailboats that have planned payload with electric and electronics, and one of them is equipped with an electrical engine that may eventually help with the sailboat propulsion. Our current results show that it is possible to augment the system confidence level for the potential energy that can be harvested from the environment and the remaining energy stored, optimizing the energy usage of autonomous vehicles and improving their energy robustness.

## 1 Introduction

Autonomous robots are machines that have embedded systems with some specific purpose, which depends on the application. Nonetheless, in general, they have computational and physical resource restrictions (Almeida, 2016), being the energy performance one of the main issues to be accounted for when developing such machines (Aldegheri et al., 2018). Surface aquatic robotic (ASV or USV) and submersible (AUV) vehicles allow human beings to explore the ocean in innovative ways, with less cost, greater efficiency, and reducing risks inherent to marine operations, quickly following its natural course towards its ultimate goal: full automation for working in the ocean. In this direction, an emerging generation of devices and their systems is being designed and developed to operate independently, making decisions during operation, without direct control of a human operator.

Nevertheless, there are several cases where energy autonomy is still a big issue, contrasting with its reliability in the face of day-to-day missions (Alaieri and Vellino, 2016). Energy autonomy refers to the robotic agent’s ability to maintain itself in a viable state for long periods, or as necessary. Its behavior must be always stable in such a way that it does not lack any vital resources. For example, in some situations, it must not exceed some limit of energy consumption. Until recently, autonomy has been always approached from a computing perspective. For example, consider the case of a battery-operated robot that is released to perform its task without outside intervention. When the task is completed or when the battery charge decreases, the robot returns to a base for recharging and/or further instructions. Thus, in this case, only certain aspects of robot behavior can be considered autonomous, for example, computational and control decisions. On the other hand, without a human in the *loop*, this kind of robot would not be able to replenish its energy to perform the task. In the case of this work, the robot should be long-running, for weeks or even months without intervention, as it will be explained further. So, the human in the *loop* is not possible and all energy management should be done by the robot system itself.

Hence, in this paper, we aim to introduce a novel energy estimation and management process, based on the Restricted Boltzmann Machine (RBM). An RBM is a stochastic network that can be used for representing undirected generative models that use a layer of hidden variables to model a distribution that has as input a set of visible variables Larochelle and Bengio (2008). RBM are widely used to compose deep belief networks (DBN) extracting characteristics from a dataset through unsupervised training (Hinton and Salakhutdinov, 2006). As it will be explained further in Subsection 2.3, the network used in this work has an initial layer with 6 neurons that, after normalization, gets to 55 neurons in the visible units and at least 55 neurons in the hidden unit. The main strategy here is to use this approach aiming at finding a solution to the distribution of energy consumption problem with solar panels in our autonomous sailboat, the F-Boat. Our current proposal is inspired by our previous project (Júnior et al., 2013; Negreiros, 2019), whose main objective is the development of an autonomous vessel for collecting and monitoring environmental data. This is an open project, with complete documentation that can be found on our web repositories (LAICA, 2015; Negreiros, 2019). The focus of the project is to develop a long-endurance autonomous system, satisfying quality criteria for being qualified as sustainable and with environmentally friendly energy generation.

**FIGURE 1:**
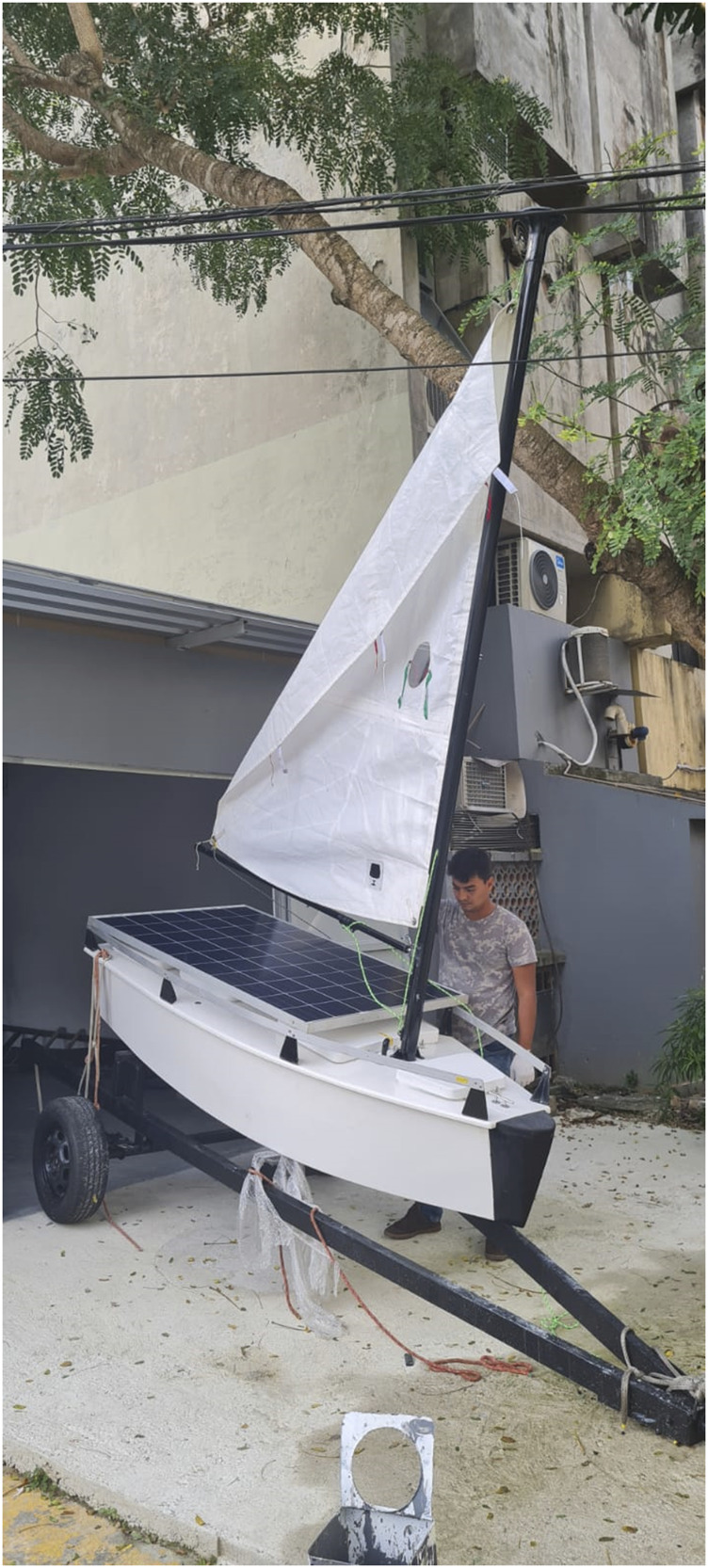
F-Boat hull with its solar panels.

The current project version named F-Boat is an evolution of previous USV projects that our research group has developed, such as the N-Boat 1 (Júnior et al., 2013) and N-Boat 2 (Negreiros, 2019). F-Boat is a twin, new version of N-Boat 2 with updated architectural design and equipment. It is also an autonomous unmanned vessel (a sailboat USV) as seen in Figure 1 With respect to the planning of missions, they can be established using our multi purpose platform regarding some restrictions. The main one is mission duration, which should be determined based on how many hours or days without solar charging the vehicle will face and if the electrical engine propulsion will be required. Considering the worst situation, with no sunlight, the theoretical endurance is 62 h without using the electrical motor (navigation only with sail and rudder). The endurance is 11 h if using the electric motor on a continuous basis, in the case of no wind situation, for example. Knowing that, it is possible to stay for a long time on the water without charging, and with the batteries recharging when necessary, which is provided by the solar panels. The two boats are self-sufficient considering their current set of electric and electronics, being satisfactory to date. However, our main issue in this paper is related to future power consumption, as the emergency electrical motor and other eventual payload devices would be embarked, which may eventually increase said consumption. In this case, planning on future available energy and defining what equipment or device will function, must be done based on an estimation of said production. In this newer version, in addition to the sensors necessary for autonomous navigation, other more dedicated sensors are used, such as the stereo camera Zed that has a 16 m range (Ortiz-Fernandez et al., 2018) for very short forward-visual sensing and a 360°camera for larger-range visual sensing. Both sensors allow the robot to perceive its immediate environment and find short-range obstacles. We notice that the depth information provided by the ZED is useful only in the short range (16 m and less). In general applications, the idea is to complement the depth information with a LIDAR, however, we have not bought it at the time that this article has been written. Nonetheless, we are working on accurate detection algorithms for the short range based on the Zed information, in order to guarantee that the boat stops when some obstacle is detected just on its front. At the moment we are also working with computer vision and AI approaches using the 360 camera, which can be applied for larger depth distances. All of these sensors generate a massive amount of data that are locally processed by an embedded processor based on an nVidia Xavier board. Part of the data is locally saved for future analysis and comparison with a simulator that has been implemented (Paravisi et al., 2019). This data can also be sent to a ground control station (GCS), which is an option for mission updates or telemetry (we use the Mission Planner for that). We initially intended to use this high-performance processing board as a kind of a single board computer (SBC), for communication with the GCS and managing all boat resources and systems, besides visual data processing. However, due to real-time restriction, we are studying the possibility of using another board as the SBC, since visual processing may complicate the real-time implementation aspects of the system. Hence, the general contribution of the project is a step forward for solving the several challenges faced when developing a sailboat robot, beginning with the boat’s architectural design and construction itself, including solutions for autonomous sailing navigation, image processing, obstacle detection, and control issues.

With this general objective of the project in mind, the energy management is treated as the specific focus of the contribution of this paper, which resides on describing reliably and ecologically correct solutions to both sailboats’ energy problems. We propose a particular solution that involves the use of *offgrid* energy production based on solar panels to maintain the more complete as possible set of components operating, such as the emergency electric motor and other actuators, processors, and cameras. Hence, it is necessary to make an intelligent use of the scarce energy resource, aiming at an autonomous, sustainable, and ecologically correct system. Having this focus in energy management, the practical contribution that we propose here is a set of rules that are implemented for setting up what are the devices that can operate given certain weather conditions to the sailboat, including emergency cases. This management is implemented by way of using a Boltzmann machine (Bu et al., 2015; Passos et al., 2020). Therefore, our main contribution is this methodology to predict energy production from solar panels in the near future, to increase the sustainability of the sailboat through better energy management and thus reducing the navigation problem induced by negative power input (consuming more than it produces). Our current results demonstrate the use of Boltzmann machine to forecast the expected future energy production as a solution to improve energy autonomy of mobile platforms.

Some theory on sailboat projects are introduced in the following Section, in which we will also describe the basics an Boltzmann Machine, that will be the intelligence approach behind the vehicle’s energy resource. Then, we provide the energy generation system followed by the use of Boltzmann Machine approach, with a experiment on energy management and our final discussions.

## 2 Background Issues Related to Autonomous Sailboat

A sailboat that intends to operate at the sea gives up important challenges that are brought by physical environmental phenomena such as waves, wind, water salinity, and temperature, among others. Most of these phenomena can be represented by dynamic variables, which is one natural alternative for building, analyzing, and comparing techniques for autonomous sailing. In order to better understand, also for design and implement a fully autonomous sailboat, it is necessary to gather some multidisciplinary contents. In this section, we get into some of these, with important issues related to the energy management, architectural design of our sailboat, and details of Boltzmann machine energy management solution.

### 2.1 Renewable Energy Sources in Sailboats

The usage of renewable sources, such as the sun, wind, tides, among others are important ways for enhancing energy autonomy in future vehicles (Dupriez-Robin et al., 2009). In this direction, the use of wind propulsion is an important solution for surface water autonomous vehicles (USV or ASV). However, to ensure that a sailboat is an electrically self-sufficient platform, since all onboard electronics require one or more energy sources, considerations must be taken regarding the total energy required and how much operating time will be spent on the missions. Rechargeable batteries are typically used as primary sources for energy storage. It is essential to consider on-site energy production using some self-sustaining model. On vessels, there are several ways to obtain energy. One of the most used is solar panels, which is an excellent alternative energy source for embedded systems in general (Raghunathan et al., 2005).

Solar panels are devices that convert energy from solar radiation into electrical energy. However, depending on factors involving the panels’ nature, such as direct radiation, hours of sunlight, and temperature, substantial variations in the amount of energy produced by these mechanisms can occur. It is necessary to use a charge controller, which stabilizes varying energy ranges. As occurs in any transformation process in nature, a part of the energy is lost. Thus, the price of this transformation is a decrease in the energy efficiency rate of the system as a whole.

It is important to mention that choosing a sail-powered vessel is a strategic and main point of long-range monitoring projects. Since we choose well-designed energy sources and also use well-defined consumption strategies (Kanellos, 2014; Khan et al., 2017; Vu et al., 2017; Letafat et al., 2020), sailboats are able to achieve full autonomy, acting independently of human beings, as long as they are programmed for the task. A fully autonomous robotic sailboat does not need to stop for recharging or refueling (Hole et al., 2016). In cases of semi-autonomy, recharging strategies during the mission must be considered and planned (Waseem et al., 2019). The architectural design adopted in our sailboat is described next.

### 2.2 N-Boat and F-Boat Behavioral Architecture

**FIGURE 2:**
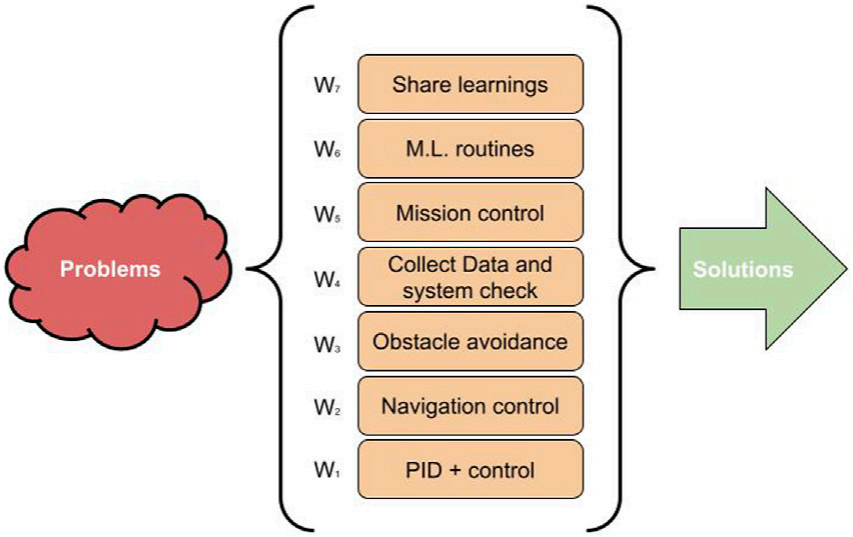
Basic architecture of N-Boat and F-Boat inspired in the subsumption architecture.

As aforementioned in the Introduction, F-Boat is an upgrade from the previous autonomous sailing boat called N-Boat. Both of them are implemented inspired on the behavioral architecture namely subsumption (Brooks, 1986), which constantly processes and executes routines of the different layers. With this approach, the basic actuation and sensing commands never cease to be executed. The basic idea of the architecture is that more basic and instinctive behaviors controls (robot survival) prevail over more sophisticated and unnecessary behaviors. In order for this to be orchestrated by a resource management algorithm, each behavior has a weight, allowing them to be ranked. It is noteworthy that behaviors below a given behavior are not suppressed, however, the behaviors above can be suppressed. Figure 2 illustrates the implementation of this approach. For example: *Navigation control* has a more critical processing weight than *Obstacle avoidance*. Thus, in extreme cases, the *Obstacle avoidance* behavior routine is suppressed by the processing of *Navigation control* routine. But it is important to understand that even in this case the *PID + control* behavior is still processed because it has even more priority.

**FIGURE 3:**
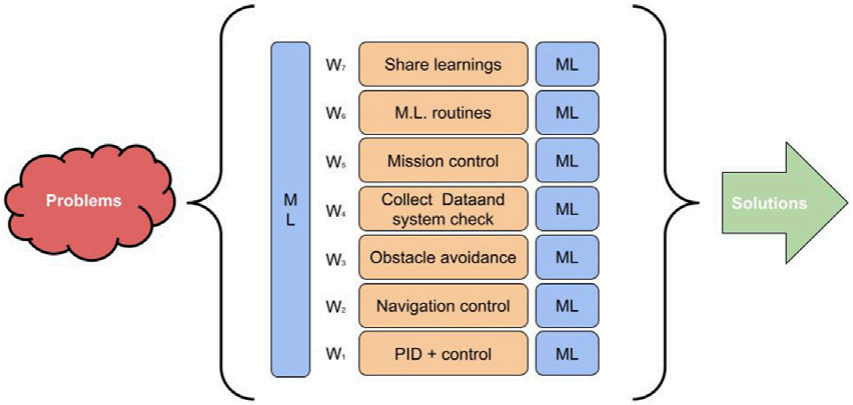
Basic architecture combined with machine learning.

The architecture represented in Figure 2 shows weights for each behavior, on the left. These weights should be constantly adjusted to adapt in real-time to the environment changes. The machine learning model, which is inserted in the left of Figure 3, is responsible for this update. Nonetheless, as the marine environment (wind, tides, weather, swells, among others) is highly dynamic, there is a great need for the weights of these behaviors to quickly adapt. Furthermore, each behavior can use machine learning, in its own context, to improve its performance. As illustrated in the blue boxes next to each behavior. For example, in the rudder control, the P, I, and D gains can be adjusted by using the ML. In cases where regular algorithms are not able to perform this task in the required time, the option of using TEDA-Cloud Bezerra et al. (2016) is listed as an alternative. For instance, if data processing can be made available online, it is possible to establish a window in which the signals can be processed with a greater degree of dynamicity (Bezerra et al., 2016).

We notice that, with current embedded computing resources, there is the possibility that behaviors can be computed at the same time. Since many control cards already have more than one physical core in the processor and chips or GPU cards. Therefore, it is quite plausible that this architecture can delegate more than one processing at the same time, which means that is possible for the boat to compute the processing of an obstacle avoidance while analyzing the processing of a payload, or the running energy management system, based on the Boltzmann machine.

The integration of the energy management system based on the Boltzmann machine as a behavior in our basic sailboat control architecture allows us to implement the energy-saving strategy. Since the behaviors also have energy consumption grades, routines that consume energy may be suppressed. It is important to mention that our machine learning implementations allows to predicting these cases, minimizing the situations where these events may happen. The behaviors that can be turned off should be above the energy management system and behaviors that can not be turned off should be below it.

### 2.3 Restricted Boltzmann Machine

The data classification problem is intrinsically related to the recognition of patterns and regularities in a given database. In the context of learning systems, classifying data is considered a supervised problem. However, unsupervised approaches, such as the restricted Boltzmann machine (Smolensky, 1986; Hinton, 2002) and autoencoders (Bourlard and Kamp, 1988), have been applied as feature extraction tools to feed supervised algorithms such as artificial neural networks (Haykin, 1999). Thus, semi-supervised techniques emerge, which have gained prominence in recent years.

The restricted Boltzmann machine (RBM) is a stochastic network widely used to compose deep belief networks (DBN) (Hinton and Salakhutdinov, 2006). RBM can extract characteristics from a dataset through unsupervised training. Due to this, approaches that use RBMs to compose a DBN were developed as the first stage of a classifier based on artificial neural networks (Salama et al., 2010; Tamilselvan and Wang, 2013).

The restricted Boltzmann machine (Smolensky, 1986; Hinton, 2002) is essentially a stochastic network consisting of two layers: visible and hidden. The visible units layer represents the observed data and is connected to the hidden layer, which in turn, must learn to extract characteristics from this data (Memisevic and Hinton, 2010). Originally, RBM was developed for binary data, both in the visible layer and the hidden layer. This approach is known as Bernoulli-Bernoulli RBM (BBRBM). Since there are problems where it is necessary to process other types of data, Hilton and Salakhutdinov (Hinton and Salakhutdinov, 2006) proposed the Gaussian-Bernoulli RBM (GBRBM), which uses a normal distribution to model the visible layer neurons. In this section, the basic concepts related to the GBRBM approach will be described.

**FIGURE 4:**
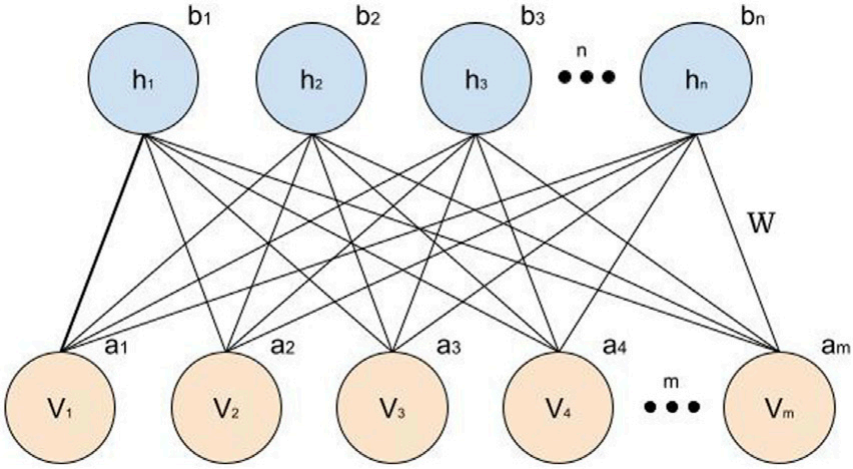
A simple Restricted Boltzmann Machine architecture.

In RBM, the connections between neurons are bidirectional and symmetrical. This means that there is information traffic in both directions of the network. Furthermore, to simplify the inference process, neurons from the same layer are not connected to each other. Therefore, there is only a connection between neurons from different layers, so that the machine is restricted. Figure 4 shows an RBM with M neurons in the visible layer (*v*_1_, …, *v*_*m*_), n neurons in the hidden layer (*h*_1_, …, *h*_*n*_), where (*a*_1_, …, *a*_*m*_) and (*b*_1_, …, *b*_*n*_) are the bias vectors and *W* corresponds to the connection weight matrix. From here to the end of Section 2, the set (*W*, *a*, *b*) will be called *θ*.

$$p\left(v,h;\theta \right)=\frac{e^{-E\left(v,h;\theta \right)}}{\sum_{\mathrm{v,h}}e^{-E\left(v,h;\theta \right)}}$$ (1)

The RBM is an energy-based model, with the joint probability distribution of the configuration (v,h) being described by:

$$E\left(v,h;\theta \right)=\overset{m}{\underset{i=1}{\sum }}\frac{{\left(v_{i}-a_{i}\right)}^{2}}{2\sigma_{i}^{2}}-\overset{n}{\underset{j=1}{\sum }}b_{j}h_{j}\overset{m,n}{\underset{i,\;j=1}{\sum }}\frac{\left(v_{i}\right)}{\sigma^{2}}h_{j}w_{i},j$$ (2)

As the RBM is restricted, it does not have neuron connections between the same layer, the probability distributions of h given v and v given h are described by Equations 3, 4, respectively.

$$p\left(h|v;\theta \right)=\underset{j}{\prod }p\left(h_{j}|v\right)$$ (3)

$$p\left(v|h;\theta \right)=\underset{i}{\prod }p\left(v_{i}|h\right)$$ (4)

The Restricted Bolztmann Machine that we use here is implemented using Python, with the Numpy, Keras, and Sklearn library help. After training, this network is able to predict the future vessel’s dynamic consumption 24 h ahead. Here, we use data collected from previous missions by the F-Boat’s predecessor vessel (N-Boat), as the sensors provided data on wind position and intensity, tides, vessel instantaneous energy consumption, sail position, and rudder position. This information is used as input to the neural network, which begins its learning process by processing these data with a bias lower than 0.01%.

## 3 Similar Projects (State of the Art)

Literature related to nautical autonomous vehicles is scarce when compared to other types of autonomous vehicles. At this point, we present the most relevant works related to the research topics of this one, mainly for comparison. As organizational criteria, all comparisons are described in Table 1 and their comments can be found in the following text. Notice that other types of vehicles appear in the table besides unmanned surface vehicles (USV), including autonomous underwater vehicles (AUV), and autonomous vehicles (AV).

**TABLE 1:** Strictly related works.

Title	Vehicles	Focus	Citation
Design of a Battery Carrying Barge	USV Sailboat	Energy endurance	Liang et al. (2021)
for Enhancing Autonomous Sailboat’s			
Endurance Capacity			
Design and Energy Consumption	USV Sailboat	Energy optimization	Ou et al. (2021)
Optimization of an Automatic			
Hybrid Sailboat			
Unmanned Surface Vehicle Simulator	USV Sailboat	Sailboat simulation	Paravisi et al. (2019a)
with Realistic Environmental Disturbances			
Offshore Sensing SailBuoy	USV Sailboat	Long endurance	Sailbuoy. (2018)
Unmanned Surface Vessel			
		surface vehicle	
Autonomous Sailboat Navigation	USV Sailboat	Many	Stelzer and Jafarmadar. (2007)
Routing and course control of an autonomous sailboat	USV Sailboat	Trace efficient routes	Saoud et al. (2015)
		using PRM-Dijkstra	
High-Level Path Planning for an	USV Sailboat	Sailboat navigation	Silva Junior et al. (2020)
Autonomous Sailboat Robot			
Using Q-Learning			
A Behavior-Based Architecture	General boats	Describe methodology	Olenderski et al. (2006)
for Realistic Autonomous			
Ship Control			
	USV		
An experimental comparison	AUV Submarine	Compare architectures	Byrnes et al. (1992)
of hierarchical and			
subsumption software architec-			
tures for control of an auto =			
nomous underwater vehicle			
Reinforcement Learning in a	AUV submarine	Controle de submarinos	Frost et al. (2015)
Behaviour-Based Control			
Architecture for Marine			
Archaeology			
Control architectures for	AUV Submarine	Survey and control of AUVs	Valavanis et al. (1997)
autonomous underwater			
vehicles			
A Hybrid Control Architecture	AUV Robot Fish	Collaborative control	Liu et al. (2006)
for Autonomous Robotic Fish			
		between fish robots	
Functional system architectures	AV Cars	Survey and car control	Taş et al. (2016)
towards fully automated driving			
Development of Autonomous	AV Cars	Car control	Jo et al. (2015)
Car—Part II: A case Study on the			
Implementation of an Autonomous			
Driving System Based on			
Distributed Architecture			
V-stability Based Control	USV Sailboat	Energy saving	Sun et al. (2021)
for Energy-saving Towards			
Long Range Sailing			

Although the Sailbuoy team (Sailbuoy, 2018) is known to be the first sailboat that completed the Microtransat challenge (Microtransat, 2021) (June 2018), the achievement was not fully autonomous, being remotely controlled at some parts of the cross. Even still, it has proven to be a robust platform, staying for months at sea transmitting and receiving data. The authors point that their solution can be used in applications for measuring ocean parameters (Hole et al., 2016), tracking oil spills, or as a communication relaying station.

Competitions are an important source of references in sailing robotics, as occurs in other robotic fields such as the world robot soccer competition (Robocup). Examples of international competitions related to robotic sailboats are the *World Robotic Sailing Championship (WRSC)*, derived from the *Microtransat Challenge*, which is a competition between autonomous sailboats aiming to cross the Atlantic Ocean. Other sailboats from Table 1 (Stelzer and Jafarmadar, 2007) and also from the literature (Alves and Cruz, 2008; Stelzer, 2013; Dahl et al., 2015) were developed to compete in this challenge.

Further, we select from Table 1 three examples of works dealing with energy management in autonomous sailboats. The first one (Sun et al., 2021) deals with energy control methods for saving energy by improving the navigation system. The second (Liang et al., 2021) tries to increase energy autonomy by changing the hull structure to carry more battery packs. The third and last topic (Ou et al., 2021) is about optimizing energy by improving the use of the motor in hybrid sailboats (sail and engine).

Finally, besides having the two twin sailboats constructed, we also consider using another simulation environment that we have built based on the N-Boat specifications (last item of Table 1) (Dos Santos and Goncalves, 2019; Paravisi et al., 2019). By using simulators it is possible to customize the same variables for different vehicles, as often as necessary. Simulated environments are a viable startup testbed that provides an initial performance for sailboat systems, tested in multiple environments, due to the aforementioned particularities and because of the number of scenarios that can be considered. For example, the Boltzmann machine can be implemented and tested in this environment, before practical implementation.

## 4 Power Generation System

Despite converting wind into kinetic movement, electrical energy is still required for the instrumentation that allows autonomous sailing, which is the emergency and maneuvering electric propulsion engine, the rudder and sail actuators, the onboard computer, sensors, and the payload. Currently, the N-Boat and F-Boat power generation systems are purely fed by solar panels and a bank of nautical batteries. Details of this generation, storage and other alternatives will be treated in the next sections. This current generation is what is needed for long-term missions (Boas et al., 2016), which do not use the electric propulsion engine constantly. The main hypothesis of this work is to allow the use of the propulsion engine, without compromising the boat’s energy supply in these missions. Therefore, it is necessary to use a strategy that allows, at certain times, under certain circumstances, its use during short a period of time.

### 4.1 Energy Generation Approaches

**FIGURE 5:**
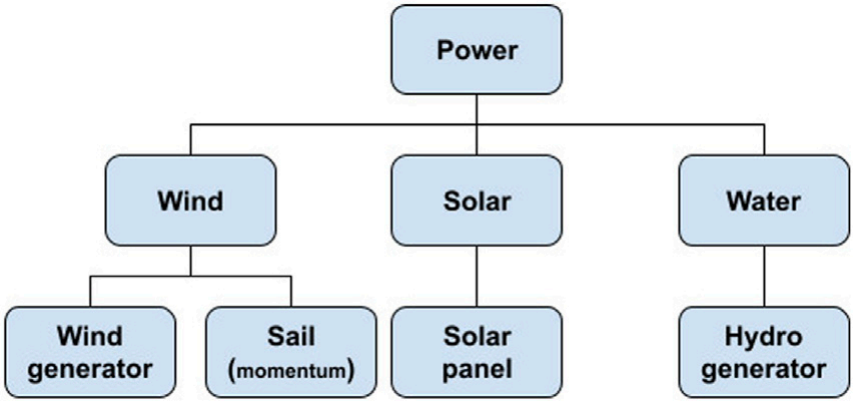
Energy generation approaches.

Self-sufficient autonomous vehicles can generate energy in several ways. In this work, we are classifying the sources into renewable (ecological) and non-renewable. The renewable correspond to Hydraulic, wind, solar, geothermal, marine, biomass and biogas. The non-renewable are oil, natural gas, coal and nuclear. Although there are many possible sources to be shipped, few are feasible, those illustrated below in Figure 5.

Renewable sources, in addition to being ecologically correct, allow for long-term missions. Several studies (Wang et al., 2008; Waseem et al., 2019) demonstrate that this alternative ends up being one of the best choices, but requires strategies that guarantee a positive energy balance at the end. Since propulsion is the main energy consumption item of autonomous vehicles, there is a notable increase in energy autonomy between sailboats and other types of autonomous vehicles that are propelled by engines.

**FIGURE 6:**
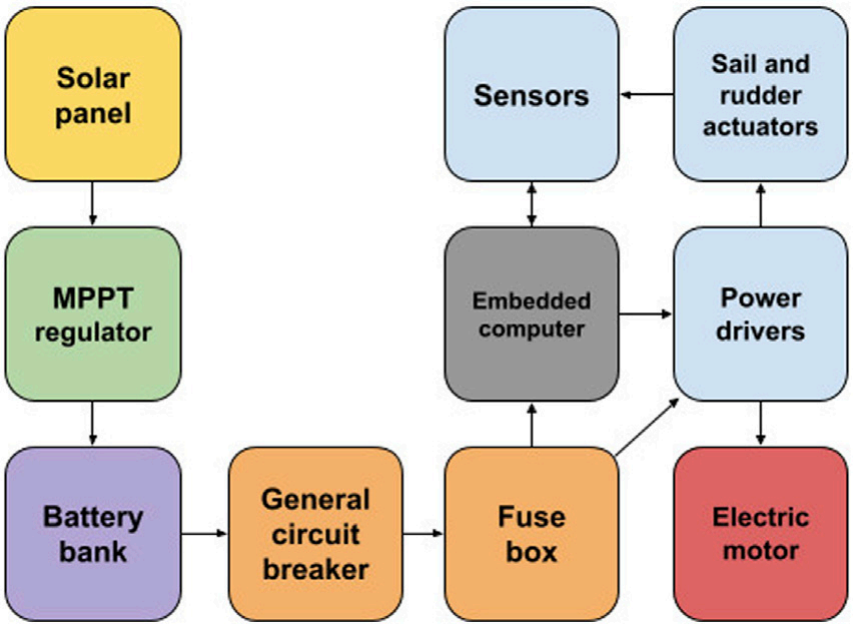
F-boat’s current energy generation model.

For a better hardware architecture understanding, we present the solution based on the N-Boat and F-Boat models. Figure Figure 6 illustrates all components that generate or consumes some type of energy in these vessels. An important point is an electric motor, which, as will be shown later on, its continuous use can compromise the entire vehicle’s autonomy. Therefore, the solution needs a strategy that only allows its use in special moments, such as poor wind conditions, return to base, emergency maneuvers, among other situations.

Our model uses a solar panel for electricity production. In the future, there is the possibility of using wind power generation and/or a mini-hydro generator. Solar production has some advantages and disadvantages. The main advantage is that it corresponds to the most efficient in terms of cost, generation, extreme weather conditions resistance, and ease of installation. However, there is a lack of moments where there is no source, such as nighttime or bad weather conditions. Another drawback is the large deck area required.

**FIGURE 7:**
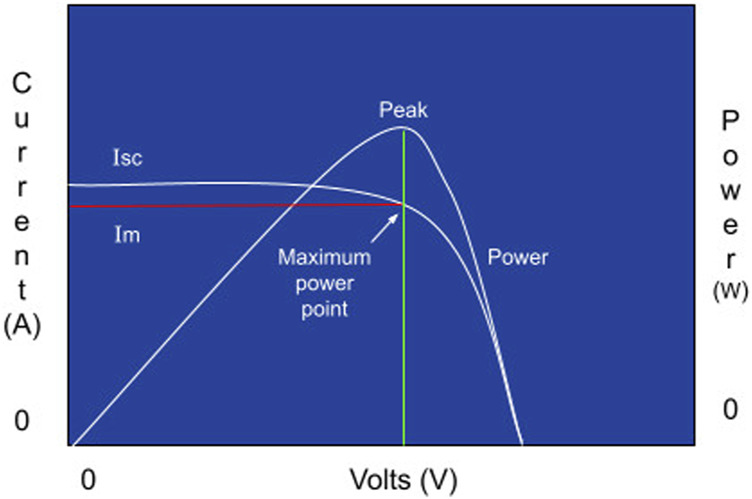
Current × Voltage curve for a photovoltaic panel.

In the present project, we adopted a panel with the current-voltage graph shown in Figure 7.

The generation of electrical energy in a photovoltaic arrangement is intermittent and is strongly determined by cloudiness and temperature. These factors cause the operating point, which leads to the extraction of maximum power from the photovoltaic array to change constantly. Thus, tracking this Maximum Power Point (MPP) continuously is a way to ensure greater efficiency in energy conversion, as can be seen in the figure Figure 7.

To control the battery charge, a charger controller is necessary. This equipment can find the perfect current and voltage ratio, charging the battery bank with maximum efficiency.

PWM, which stands for Pulse Width Modulation, is a charger controller that keeps a battery fully charged through high-frequency voltage pulses. Thus, this driver allows to check the battery charge status and adjust the sent pulses. This type is more used in the market as it has a lower price than an MPPT driver.

MPPT stands for maximum power point tracking and is a charger controller that looks for the best power point of the module or solar panel. Enabling the system to make the maximum power the panel has to offer and also can monitor energy production and reduce system losses. This type of driver is more expensive than the previous one, but it promotes greater efficiency than the PWM controller.

**FIGURE 8:**
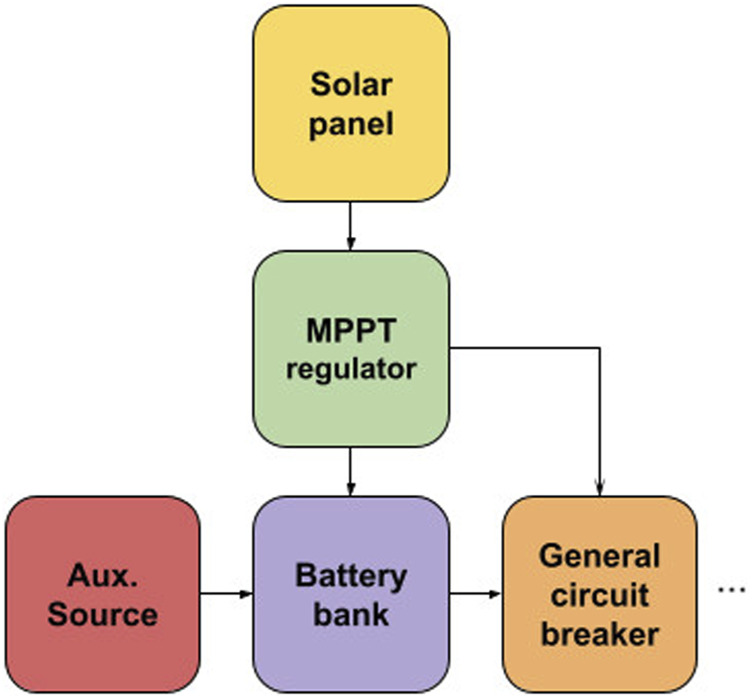
MPPT regulator connected directly to power distribution.

In Figure Figure 8, it is possible to see a schematic diagram that reflects an alternative configuration on the N-Boat and F-Boat. For reasons of energy efficiency, the MPPT load driver is directly connected to the power distribution. As the electronic arrangement was designed with maximum system robustness and reliability, power output is connected directly from the battery bank, being much safer than installing through the charge controller mentioned beforehand. Therefore, the boat can still be powered by the battery bank with a remaining charge. Here we also mention the necessity of using a general circuit breaker and a fuse box (with one for each compartment) guaranteeing maximum safety.

### 4.2 Electric and Electronic Components

**FIGURE 9:**
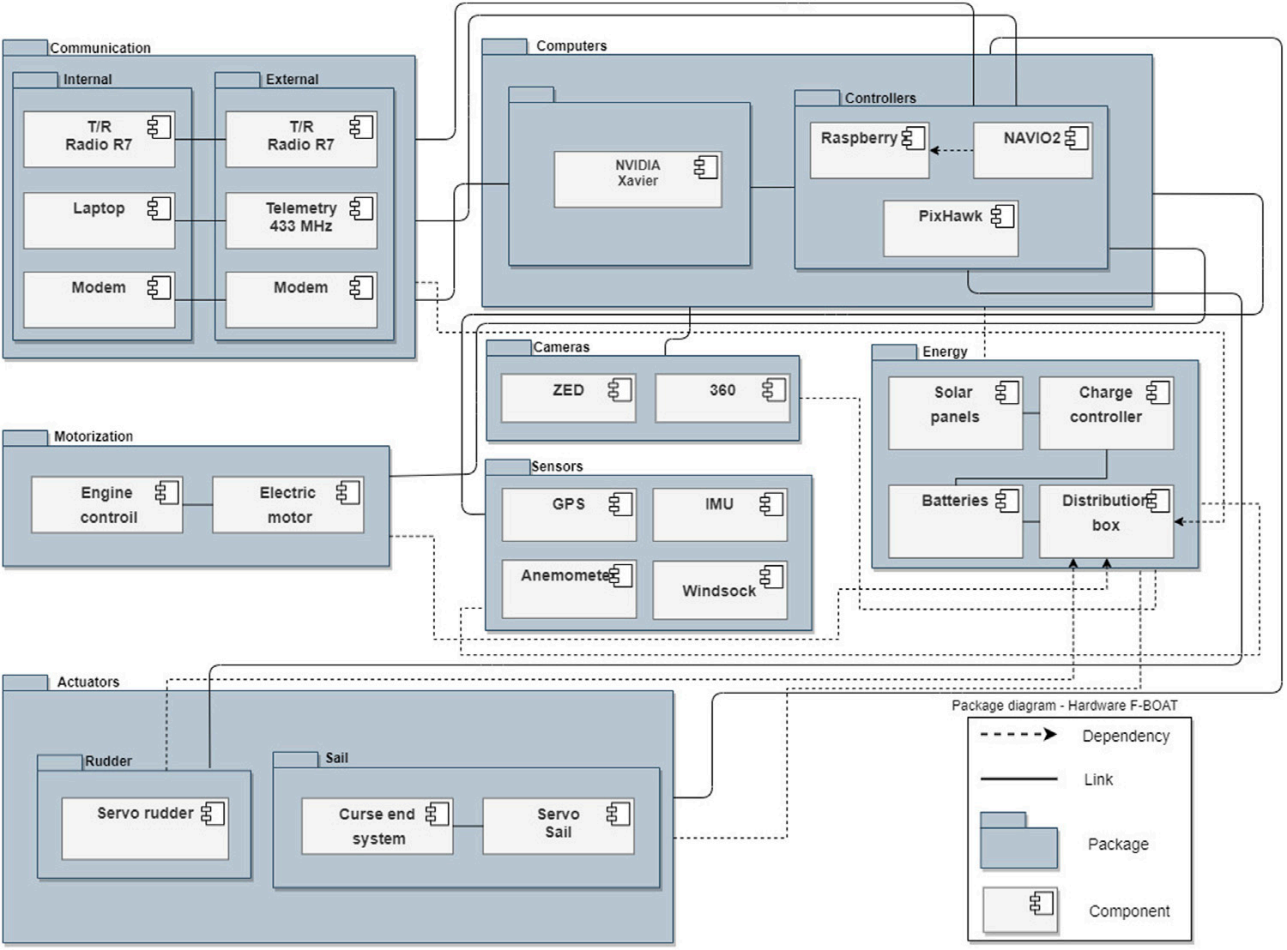
F-Boat’s package flow diagram.

In Figure 9 it is possible to visualize the F-Boat packages diagram. It features all of the vehicle’s electrical and electronic components in a structured way, including also related documents, classes, diagrams, and packages. Each package is better described below.

The energy package, which is the main focus of this work, contains the entire set of equipment responsible for the generation, storage, and distribution of energy throughout the vessel. The sail package contains the components responsible for controlling the sail angular position and the rudder, as its name suggests, involves the components responsible for controlling the vessel’s rudder. Computers are the devices responsible for all planning and data processing on the vessel. The communication package contains all components related to both internal (between all internal components of the sailboat) and external (communication with the shore base) communication. Motorization package relates all necessary hardware to motorize the sailboat, used in specific situations. Sensors are responsible for capturing the necessary data for both monitoring and general movement of the sailboat, working together with the cameras that are used for image acquisition, responsible for the computer vision tasks of the vessel.

### 4.3 System Consumption

Using solar panels, the production is enough to keep, during a short time, the sailboat system working without a lack of energy. Of course, without using the electric propulsion motor. The Boltzmann machine will be used for days when there is no full sunlight and/or massive use of electric propulsion is required. In these cases, the boat will need to turn off some modules and behaviors for it to survive these moments. This same strategy could be used to further increase their autonomy, if necessary, as will be described later in this work.

As each battery has 111 A/h and the boat has 4 batteries of these embarked, the total of available amperage is 444 A/h. In practice, this may go to lower values, as these batteries may not be 100% charged, addicted, or the battery life can vary from its use and falling. Anyway, it is possible to assume, for a theoretical environment, that this available power is sufficient for the tests. Considering the consumption labeled in each equipment manual, as shown in Table 2.

**TABLE 2:** Calculated energy consumption.

**N°**	Description	Fixed/Variable	Consumption
1	Hardware	Fixed	2.0 A/h
2	Sensors	Fixed	0.2 A/h
3	Actuators	Fixed	0.9 A/h
4	Cameras	Fixed	1.0 A/h
5	Outboard engine	Variable	30.0 A/h
6	Sail winch	Variable	3.0 A/h

The same table (Table 2)shows the components expected energy consumption, which is informed by the datasheet or by laboratory tests carried out in previous experiments. Notice that, in the table, some of the items are labeled as fixed or variable. This is because some of them stay turned on throughout the entire mission, so their consumption is often identical to reported in the datasheet. However, other components have a consumption relative to their use. Such as the sail winch, which is only used when it is necessary to trim the sail. Taking into account the minutes that were turned on and relating it to the total consumption hour, so that it is possible to estimate an initial of the vessel’s total energy consumption.

## 5 Energy Management Experiments With RBM

Even though sailboats use wind as their main source of energy for their movement, electrical energy is still necessary for directing the sail and the rudder and also for their other components. As the production and storage of this energy resource is limited and scarce, therefore, a system that distributes energy efficiently and safely inside the vehicle is necessary. Besides, a strategy is essential to make intelligent use of this resource. With that in mind, we designed our vessel so that in the case that the solar panels stop producing energy, it is still possible to make severe use of the battery banks during the next 48 h, without completely discharging. This happens at night, for example (a 12 h period, considering Natal, Brazil). This is to say that the boat would still have remaining energy when the solar panels start recharging again. If the recharging does not happen accordingly, the boat will completely stop all systems.

That alone enables an ability to perform long missions, in case of regions with restrictions on the periods of day and night. Besides, protocols and consumption strategies were programmed, checking this energy consumption process. For example, on a given mission, it is possible to stay closer to the theoretical route, at a higher energy cost. However, it is known that performing fewer maneuvers reduces the sailboat consumption, but the actual accuracy on the theoretical route would also decrease. Another example would be to make little use of image processing in certain open sea locations. Leaving this feature to places with already pre-mapped obstacles or in places full of ships traffic. In addition to the embedded technology, all of this raw and processed data is sent to a command base ashore or a nearby support vessel. These data are displayed *via* a user-friendly platform that allows data tracking, route changes, strategy changes, and manual control of the sailboat for extreme situations. Besides these exemplified strategies can and should be taken into account, they are not the main subject of this work, as here we discuss the possibility energy estimation. Actually, the application of solar panels systems for ships depends on many factors mainly: 1) Solar radiation availability in ship’s operation areas; 2) existence of sufficient and adequate deck area to accommodate the solar panels; and 3) techno-economic efficiency of a solar panels system that includes energy efficiency, fuel oil rates, and investment costs.

Hence, in order to simulate the behavior of the system for the sailboats, we implemented and tested it through a Boltzmann Machine using data collected from the electrical and electronic components of the N-Boat hardware architecture, such as described above. The implemented system aims to show a solution to find a better way to use the distribution of energy consumption with solar panels in our autonomous sailboat F-Boat, which contains a saving/rescuing engine. Through sensors and data obtained directly from the charge controller, the proposed system monitors and manages component power control through a relay system. The system also connects to two modules: computers, where it can change the energy operation of some of its components depending on the current state of charging the batteries, and to the vessel’s communication package, which can send data to the external monitoring application.

We use the Boltzmann machine neural network with restrictions to predict the vessel’s energy consumption in a 24-h range. For this forecast, we designed the network to read data provided by sensors on the vessel itself, thus using external natural phenomena, winds, tides, lighting density, angle of incidence of sunlight. As navigation aids, we can predict the vessel’s energy consumption with and without the use of solar panels.

The Restricted Boltzmann machine neural network is fully developed using Anaconda, Spyder 5 platform which is an open-source cross-platform integrated development environment for scientific programming in the Python language. This development is aided by Numpy (2021), Keras (2021), TensorFlow (2021) and Scikit-Learn (2021) libraries, which are open source neural network libraries written in Python. They are capable of running on top of TensorFlow, Microsoft Cognitive Toolkit, R, Theano, or PlaidML. These tools are designed to allow quick experimentation with deep neural networks, focusing on being easy to use, modular and extensible.

Hence, we implemented a neural network code using standardized techniques to facilitate the implementation of the learning process with the network. The Boltzmann machine has libraries already created and tested by other researchers, so we can focus on information and data collected in the research, by using these standard techniques. Most of them have their repository on the Github site and in some cases, such as Tensor-flow, it has its own library for research with official forums of participating developers their difficulties and successes in designing architectures using these techniques. Our network has an initial layer with 6 neurons that receive input tide data, wind speed and direction, vessel battery voltage, average motor consumption, and consumption of the actuators. After normalization, we get 55 neurons invisible units and more than 55 neurons in hidden units, connected similarly to the network presented in Figure 4, which process the data in 7 h and 28 min with 4,903 iterations of epochs. As result, we obtain an estimated consumption of the vessel in the range of 24 h.

**FIGURE 10:**
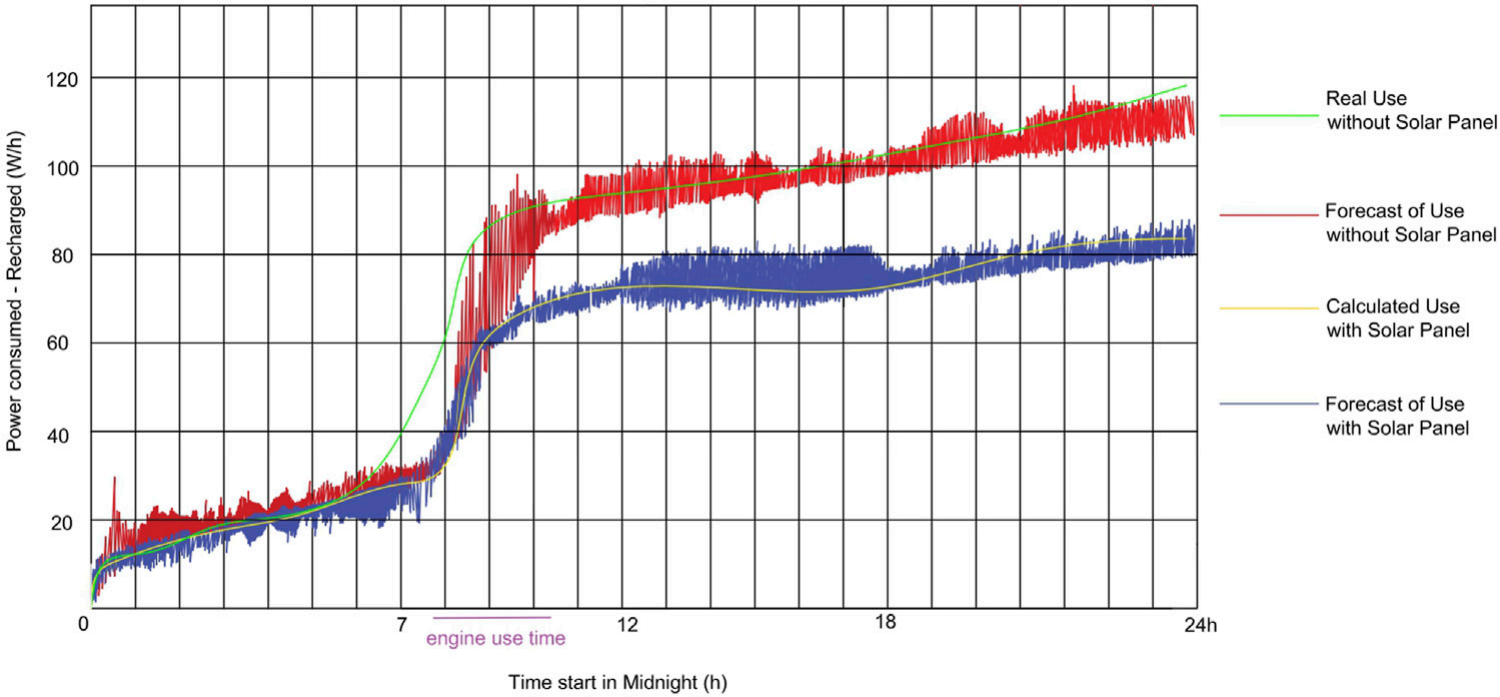
Results for estimated consumption of the sailboat on a 24 h duration simulation, with data extracted from the N-Boat sailing experiment.

The graphs in Figure 10 show the results generated by our experiments. The green line is the consumption data that we calculate by taking data from the N-Boat’s real electrical and electronic components (Table 2). Notice that it is not the online (real-time) consumed value of a true mission, however it is very close to it as we use the real values of the equipment and devices consumption to calculate it. The yellow line is the data calculated for the N-Boat if using a solar panel. The red line is RBM’s predictions without a solar panel, which were later placed under the real consumption data (calculated from N-Boat electric and electronics) to understand if it was correctly estimating the real value. The blue line is the RBM estimates with the solar panel data.

We notice that for the experiments shown in Figure 10, we estimated the recharge of the panels in operation by way of using mathematical calculations based on the MPPT charger controller actual parameters. Nowadays, we are already working on the implementation of sensors for collection of this kind of data in the newer version of our project, the F-Boat. So, the real consumption value will soon have the real value given by these sensors and the estimated value of consumption with the solar panel given by using the neural network.

### 5.1 Results Discussion

With the collected data, our objective is to define the success rate of a mission related to energy failures. Using maritime currents, wind speed, solar luminance rate, among others, and measuring how much natural energy can be transformed into mechanical energy, we can estimate the success of a mission. We chose to use a neural network so that it can re-establish the calculations in case a natural phenomenon occurs suddenly, a tide change or a storm that changes the wind or reduces the sunlight, for example. Some questions appear that are already answered or that we will work on in the short time. The first one is what are we looking at here? We are looking for a system that can be used to eventually help save energy and keep the sailboat alive (running). Second question is how far ahead can we predict energy consumption? This is one of the parameters that serves as input to the Boltzmann machine. We should guarantee for this time the minimum of 48 h as set in the N-Boat and F-Boat initial design. With this time, some rescuing action can be taken.

Yet the model needs to train for 7 h and 28 min in order to generate a useful output, however with transfer learning this time can be diminished. In practice, we believe that the mechanism can be implemented in the SBC embarked, which are nVidia boards Xavier, for online learning and changing the output parameters in certain situations. We believe that this will produce a usable system which can give predictions in real time. At this time the mechanism is only suitable for offline use. Thus, what we are generating is not a forecast or prediction of energy consumption, but a model of actual data given by N-Boat (our first sailboat) data. This simulation uses data from a real mission that it performed in Natal, Brazil, in normal weather conditions. In such an experiment, the boat basically used sail and rudder to perform some maneuverings. A last question is how would we use this? This is not fully operating in our real sailboats yet. However, from these initial experiments and tests we could devise a way for the sailboat to manage without human intervention and decide if certain equipment can be turned on or not during a long running mission. Next step, in a very short time, is to put all of these running inside the F-Boat, which is operational at Guanabara bay, Niteroi, Brazil (https://youtu.be/mvJdl09Jazo).

## 6 Conclusion and Future Work

We verified the use of the Boltzmann Machine as a prediction tool for helping the management of the energy in our autonomous sailing boats projects, and achieved some expected responses. The association between renewable and continuous energy generation with an energy management strategy using the Boltzmann machine indicates positively in this direction. Our results show that using artificial intelligence is a possible direction of research towards defining a strategy for energy monitoring, in order to further suggest decisions such as sail movement, the best path to the target and the correct start/stop times for the electric propulsion engine.

Besides the results obtained in the simulation using the Boltzmann machine showed some evidence of a solution to the intelligent energy use problem in the sailboat, a series of future work are still necessary in order to further provide better results on this subject. Actually, more work is already planned to be done on this project in order to improve the current one, such as the introduction of other forms of sustainable energy generation (wind and hydro generation), system monitoring through specific sensors for the entire solar panels, providing more precise information related to charging rate, battery bank charge, battery temperature, charge controller errors, among others. Still, a more effective survey of component energy consumption through bench and field testing in various work modes is required.

## Data Availability

The original contributions presented in the study are included in the article/Supplementary Material, further inquiries can be directed to the corresponding author.
